# Does Hair Dye Use Increase the Risk of Breast Cancer? A Population-Based Case-Control Study of Finnish Women

**DOI:** 10.1371/journal.pone.0135190

**Published:** 2015-08-11

**Authors:** Sanna Heikkinen, Janne Pitkäniemi, Tytti Sarkeala, Nea Malila, Markku Koskenvuo

**Affiliations:** 1 Finnish Cancer Registry, Institute for Statistical and Epidemiological Cancer Research, Unioninkatu 22, FI-00130, Helsinki, Finland; 2 University of Helsinki, Hjelt Institute, Department of Public Health, PO Box 41 (Mannerheimintie 172), FI-00014 University of Helsinki, Helsinki, Finland; 3 School of Health Sciences, University of Tampere, FI-33014 University of Tampere, Tampere, Finland; Kuopio University Hospital, FINLAND

## Abstract

**Introduction:**

Role of hair dyes in the etiology of breast cancer has occasionally raised concern but previous research has concluded with mixed results. Remnants of prohibited aromatic amines have been found in many hair dye products, and elevated levels of DNA-adducts of these amines have been detected from breast epithelial cells of hair dye users. However, the IARC working group has concluded that there is inadequate evidence for carcinogenicity of personal hair dye use and limited evidence in experimental animals for carcinogenicity of hair colorants.

**Material and Methods:**

We investigated whether the use of hair dyes is associated with breast cancer risk in women. The study design was a retrospective population-based case-control study in Finland, with a self-administered questionnaire from 6,567 breast cancer patients, aged 22–60 years and diagnosed in 2000–2007, and their 21,598 matched controls. We report odds ratios (OR) with 95% confidence interval (95% CI) from a conditional logistic regression model applied to the frequency matched sets of cases and controls. Bias-adjusted odds ratios from the sensitivity analysis are also presented.

**Results:**

After adjusting for potential confounders, the odds of breast cancer increased by 23% (OR: 1.23, 95% CI: 1.11–1.36) among women who used hair dyes compared to those who did not. In women born before 1950 an increase of 28% was noted (OR: 1.28, 95% CI: 1.10–1.48). We also observed a significant trend between the OR and cumulative use of hair dyes (*P*: 0.005). Bias-adjusted odds ratios varied between 1.04 and 2.50.

**Conclusions:**

Our results suggest that use of hair dyes is associated with breast cancer incidence. The impact on public health may be substantial due to vast popularity of hair coloring in modern societies. It should be noted that regardless of all efforts, a possibility of bias cannot definitively be ruled out and use of a prospective design is warranted. Based on the present results, it may be concluded however that safety of hair dyes in relation to breast cancer cannot yet be fully acknowledged and lack of external safety assessment within the cosmetics industry is of major concern.

## Introduction

It has been suggested that certain chemical compounds, especially aromatic amines that are often present in commercial hair dyes and bleaches, may play a role in the etiology of some human cancers. Research results have though been inconclusive.[1] According to the estimate of the European Commission, some 60% of women and 5–10% of men in Europe use hair dyes on average six to eight times per year [2]. Considering the extensive use of hair dyes, even a small increase in risk may have an immense impact on public health.

Hair dyes can roughly be categorized as permanent, oxidative hair dyes and semi-permanent and temporary dyes, the latter two being mainly non-oxidative[3]. Permanent hair dyes are the most commonly used. They consist of colorless dye intermediates and so called dye couplers, which are derivatives of aniline (phenyl amine) and give the specific color wanted. In the presence of hydrogen peroxide, the intermediates and couplers react with one another to form pigment molecules. Darker colors are formed by using higher concentrations of intermediates. Non-oxidative semi-permanent and temporary hair dyes include colored compounds that stain hair directly.[4,5]. Hair coloring has long traditions, dating back thousands of years. Oxidative hair dyes were introduced in the end of the 19^th^ century, before which natural dyes, such as henna dominated. In the 1970 and 1980’s, a variety of new hair dye substances were brought to the market, while at the same time concerns were raised on the safety of certain dyeing chemicals. This led to banning of several potential carcinogenic substances in the EU, but not in the US [5].

P-Phenylenediamine (PPD) is among the most commonly used hair dye compounds [4]. It is hypothesized that some of the potential carcinogenic effect of PPD is due to its contamination with 4-aminobiphenyl (4-ABP, CAS no. 92-67-1) during the production process. 4-ABP has been classified as carcinogenic to humans by the International Agency for Research on Cancer (IARC) [6]. Its use was prohibited in cosmetic products in the European Union only in 2004, even though sale to general public was forbidden already in 1989 and manufacture in 1998 [6–7]. 4-ABP is also strictly regulated e.g in the United States [8]. It is specifically of concern that despite of the legislative restrictions, 4-ABP has been found in many of the hair dye products currently on the market with concentrations above the approved limits [9,10].

The mechanism of a possible association between hair dye use and breast cancer, and most specifically of the potential tumorigenesis induced by 4-ABP, is not clear. 4-ABP has been suggested to have an ability to produce mutations in the human genome[5]. Knowing the significant etiologic role of estrogen in breast cancer, one possible pathway could also be through estrogenic activity of 4-ABP [11]. IARC working group 2008 evaluated the main route of exposure to hair dye components being dermal absorption regarding both occupational exposure as well as personal use, airborne exposure playing a smaller role. The monograph concluded that there was insufficient evidence on the carcinogenicity of hair dyes. [5].

We investigated the relationship of personal hair dye use and occurrence of breast cancer in a population-based series of some 6 800 breast cancer cases and 21 600 controls. The main objective was to determine whether the use of hair dyes is associated with the risk of breast cancer.

## Material and Methods

The study design was a retrospective, frequency matched population-based case-control study with a questionnaire conducted in 2009. Case ascertainment was done in December 2008 from the population-based, nationwide cancer registry, covering close to 100% of solid tumors [12]. All women in Finland aged 22 to 60 years and diagnosed with first in-situ or invasive breast cancer between 1^st^ January 2000 and 31^st^ December 2007 were considered eligible (N = 14 815). Age-matched controls were sampled from the central population register (N = 64 353). For the purpose of this study, re-matching by birth year was conducted to match the cases and controls in an exact ratio of 1:4 and the surplus of controls and cases were randomly excluded from the appropriate birth year cohorts.

Of the 14 815 breast cancer cases identified from the cancer registry, 1550 had died before the start of the study, leaving 13 265 cases in the sample. After re-matching the cases and controls by birth year, 10 448 women with breast cancer were left in the data. Of these, 951 were excluded due to any previous malignancy, leaving 9 537 cancer cases for the study, out of which 6567 responded to the questionnaire (69%). With respect to morphology of the cancers, 5248 (80%) were ductal carcinomas, of which 4758 (91%) invasive and 1022 (16%) lobular carcinomas, of which 1002 (98%) invasive. The remaining 4% (N = 297) included cases of medullary, mucinous, tubular, and other infrequent types of breast cancer, or cases where morphology could not be clearly specified. From all breast cancer cases in the study, 99% were based on histological confirmation.

The questionnaire Women’s Health and Use of Hormones was initially developed to address the association between the use of hormonal intrauterine device and breast cancer [13] and it also served in this study as the source of exposure information. The questionnaire was self-administered and identical for cases and controls. It mapped out a large variety of known risk factors of breast cancer, such as parity, age at first birth, family history of breast cancer, menarche age, alcohol use, body mass index (BMI) and level of education, as well as several other behavioral and lifestyle-related factors.

The exposure of primary interest in this study was the use of hair dyes. The respondents were asked to estimate the cumulative number of hair dye episodes during life, age at first use and the types of dyes used. Regarding the total number of hair dye episodes during life, the response options were categorized as: Never, 1–2 times, 3–9 times, 10–34 times, 35–89 times or 90 times or more. In a pooled analysis, women reporting using hair dyes ‘Never’ or ‘1–2 times’ in their lifetime were classified as never-users, all other categories counting as ever-users. Age at first dye was categorized as: Under 20 years of age, 20–29 years, 30–39 years, and 40 years or older. The different dye types were defined as: ‘Temporary’ = a color that rinses off at first or few washes, ‘Semi-permanent’ = a color that rinses off after several washes, ‘Permanent’ = a color that does not wash off, ‘Bleach’ = the hair was bleached before coloring and ‘Partial’ = the hair was only partially dyed, e.g. highlighted. The frequency of dyeings in each of the type-specific categories was classified as Never, Rarely, Quite Often, Often.

We report odds ratios (OR), with their 95% confidence intervals (CI) from the conditional logistic regression model applied to frequency matched study design. Potential confounding factors, including parity, age at first birth, family history of breast cancer, menarche age, use of hormonal contraceptives, physical activity, alcohol use, BMI and education were included in the multivariate adjusted model, as suggested by the step wise model search. We tested the trend in the log-ORs (dose-response) of the number of hair dye episodes and the odds of breast cancer by treating the number of episodes as a continuous variable in the logistic regression. Attributable fraction in the exposed represents the magnitude of the role of hair dye use in breast cancer risk in the exposed and was calculated with a formula presented by Greenland ((OR-1)/OR) x 100) [14]. To identify differences in the odds of breast cancer in women with different type of hair dye exposure history, results stratified by birth year cohorts are presented. Subjects with missing values in any of the covariates in the fitted model were excluded.

Owing to comprehensive cancer information from the population based cancer registry in Finland, the coverage of cancer diagnoses was close to complete [12] and the role of a possible selection/ascertainment bias was considered to be negligible. A deterministic sensitivity analysis was conducted in an effort to assess other potential sources of bias affecting the observed findings. Misclassification of the main exposure of interest (hair dye use), non-response bias with respect to hair dye use and a role of socio-economic status as an uncontrolled confounder were considered and bias-adjusted odds ratios with bias percentages are presented.

The data was analyzed using R, version 3.1.1., with packages Epi (1.1.67), car (2.0–22) and Survival (2.37–7). The sensitivity analysis was performed with Stata version 12, using the Episensi-command, as introduced by Orsini and colleagues [15].

The study was approved by the ethical committee of HUS (Helsinki and Uusimaa Health district, no. 322/E0/2007) and permission for data linkage was obtained from the National Institute of Health and Welfare (THL, former STAKES, no. 2920/605/2008, extension permission no. THL/221/5.05.00/2014). Permission to contact the patients was issued by the senior physician of the appropriate hospital. The aims and objectives of the study were explained at the cover letter of the questionnaire, and returning of the filled questionnaire was considered as an informed consent.

## Results and Discussion

As of the controls, 41 978 subjects remained in the sample after the re-matching, out of the 64 353 originally sampled. Of these 23 114 responded to the questionnaire (55%). A previous malignancy was reported by 1516 controls and these were excluded, leaving 21 598 controls in the analytical data set.

Distribution of the diagnostic age did not differ between the cases who answered to the questionnaire and those who did not, being 52 years in both groups. Median age at the time of the questionnaire was 57.5 years among the responded cases as well as controls. As expected, cases had a higher response rate (69%) than the controls (55%). Age distributions are presented in Table 1. Median diagnostic age of breast cancer cases died before the questionnaire was 51 years. Regarding morphology, 76% of these were ductal and 17% lobular carcinomas. As for staging, 76% of were metastasized cancers, in contrast to 39% of the cancers of the respondent cases.

**Table 1:** Numbers and percentages of the responded and non-responded breast cancer cases and controls and median ages of the cases at diagnosis, according to birth-year cohorts.

Birth year	Contacted (% of total) *Cases*	Contacted (% of total) *Controls*	Responded (% of total) *Cases*	Responded (% of total) *Controls*	Non-resp. (% of total) *Cases*	Non-resp. (% of total) *Controls*	Resp. rate *Cases*	Resp. rate *Controls*	Median diag. age (in resp. cases)
**Before 1950**	3504 (37%)	15 067 (37%)	2446 (37%)	7876 (36%)	1058 (36%)	7191 (38%)	70	52	58 years
**Between 1950 and 1959**	4366 (46%)	18 374 (45%)	3004 (46%)	9994 (46%)	1362 (46%)	8380 (44%)	69	54	50 years
**In or after 1960**	1667 (17%)	7021 (17%)	1117 (17%)	3728 (17%)	550 (19%)	3293 (17%)	67	53	41 years
***Total***	9537	40 462	6567	21 598	2970	18 864	69	53	NA
**Median diag. age of the cases**	52 years	NA	52 years	NA	52 years	NA	NA		NA

Prevalence of other breast cancer risk factors according to hair dye use (24 479 users and 3 316 non-users) are shown in Table 2. The popularity of hair coloring varied along the calendar time. While 84% of the women born before 1950 had ever used hair dyes, the corresponding figure among women born in or after 1960 was 92%. Hair dye users reported more often ever-use of alcohol, with only 7% of them reporting never-use, compared to never-use of 27% among the non-hair dye users. Hair dye users also smoked more often (ever-smoking of 46% vs. 29%) and were more often users of hormonal contraceptives than non-hair dye users (ever-using 76% vs. 54%), while non-hair dye users were more often nulliparous (18% vs. 10%) and single (12% vs. 7%).

**Table 2:** Selected characteristics of hair dye users and non-users.

	Hair dye users (N: 24 479)		Non-hair dye users (N: 3316)	
	*Number*	%*	*Number*	%*
**Birth cohort**				
Before 1950	8447	841	1657	161
1950–1959	11586	902	1287	102
In or after 1960	4446	923	372	83
**Alcohol use**				
Never	1781	7	887	27
Occasionally	11610	48	1544	47
≤ 3 times/week	9703	40	735	22
≥ 4 times/week	1204	5	127	4
*Missing*	*181*	*1*	*23*	*1*
**Smoking history**				
Never	12935	53	2298	69
Ever	11326	46	978	29
*Missing*	*218*	*1*	*40*	*1*
**Education**				
≤ 9 yrs (Basic)	13124	54	1893	57
10–12 yrs (High school)	2272	9	273	8
13–16 yrs (Polytech.)	5629	23	603	18
≥17 yrs (Univ.)	3316	14	520	16
*Missing*	*138*	*1*	*27*	*1*
**Past use of hormonal contraceptives**				
Never	5771	24	1501	45
Ever	18526	76	1778	54
*Missing*	*182*	*1*	*37*	*1*
**Past use of hormone treatment therapy**				
Never	14422	59	2168	65
Ever	9477	39	1037	31
*Missing*	*580*	*2*	*111*	*3*
**Parity**				
No	2455	10	607	18
Yes	21931	90	2692	81
*Missing*	*93*	*0*	*17*	*1*
**Family history of breast cancer**				
No	21151	86	2830	85
Yes	2985	12	427	13
*Missing*	*343*	*1*	*59*	*2*
**Marital status**				
Single	1606	7	395	12
Married or Common-Law				
Marriage	17884	73	2316	70
Divorced or separated	3711	15	362	11
Widowed	1185	5	224	7
*Missing*	*93*	*0*	*19*	*1*
**Body Mass Index**				
<25 kg/m^2^	10380	42	1310	40
25–29.9 kg/m^2^	8122	33	1035	31
≥30 kg/m^2^	4344	18	667	20
*Missing*	*1633*	*7*	*304*	*9*
**Physical activity**				
None	1673	7	347	10
≤2–3 times/month	5664	23	816	25
Once/week	6723	27	802	24
Several times/week	10040	41	1275	38
*Missing*	*379*	*2*	*76*	*2*

*Percentages rounded to 0 decimal and may not always add up to 100

No. of missing values as for hair dye use: 370

^1^Of those born before 1950

^2^Of those born between 1950 and 1959

^3^Of those born in or after 1960

The odds of breast cancer was significantly increased (OR 1.23, 95% CI: 1.11–1.36), when comparing ever vs. never users of hair dyes after adjusting for other risk factors (Table 3). Considering the cumulative number of hair dye episodes, the odds ratio ranged from 1.07 (1–2 dye episodes) to 1.31 (35–89 dye episodes) and a statistically significant trend was observed (*P* = 0.005). Early age at first dye (20–29) was associated with higher odds of breast cancer when compared to those started at 40 years of age or later (OR 1.14, 95% CI: 1.05–1.25). The association was not, however, seen in those with a first dye episode before the age 20 (OR 1.06, 95% CI 0.96–1.16). In a pooled estimate (starting age <30 vs. ≥30), an increase of 7% was observed (OR: 1.07, 95% CI: 1.01–1.14) among women who had started using hair dyes before the age 30.

**Table 3:** Results from the analysis of the association between hair dye use and odds of breast cancer*.

	Cases	Controls	OR Univariate	OR Multivariate
**Pooled ever-use**				
Ever	5793	18686	1.15 (1.06–1.24)	1.23 (1.11–1.36)
Never¥	702	2614	1.0	1.0
*NA*	*72*	*298*		
**Number of dyes**				
0	412	1510	1.0	1.0
1–2	290	1104	0.97 (0.83–1.13)	1.07 (0.88–1.29)
3–9	829	2737	1.11 (0.98–1.25)	1.19 (1.03–1.39)
10–34	2153	6989	1.13 (1.02–1.26)	1.28 (1.12–1.47)
35–89	1714	5305	1.18 (1.06–1.32)	1.31 (1.14–1.51)
≥ 90	1097	3655	1.11 (0.99–1.24)	1.25 (1.08–1.45)
*NA*	*72*	*298*		
***Trend***			*P* = 0.0005	*P* = 0.00006
**Age at first dye**				
< 20	1539	5212	1.00 (0.92–1.08)	1.06 (0.96–1.16)
20–29	2061	6453	1.07 (0.99–1.15)	1.14 (1.05–1.25)
30–39	1282	4171	1.03 (0.95–1.12)	1.06 (0.97–1.16)
≥ 40	1211	4017	1.0	1.0
*NA*	*474*	*1745*		
**Pooled age**				
<30	3600	11665	1.02 (0.97–1.08)	1.07 (1.01–1.14)
≥30	2493	8188	1.0	1.0
*NA*	*474*	*1745*		
***Trend***			*P* = 0.4150	*P* = 0.0196
**Temporary dye** †				
Ever	573	1726	1.22 (1.09–1.36)	1.32 (1.16–1.52)
Never	4074	12769	1.18 (1.09–1.28)	1.27 (1.14–1.41)
Non-hair dye user	702	2614	1.0	1.0
*NA*	*1218*	*4489*		
**Semi-permanent dye**				
Ever	2524	7716	1.21 (1.11–1.32)	1.31 (1.17–1.46)
Never	2646	8429	1.17 (1.07–1.27)	1.25 (1.12–1.39)
Non-hair dye user	702	2614	1.0	1.0
*NA*	*695*	*2839*		
**Permanent dye**				
Ever	3059	9769	1.17 (1.07–1.27)	1.25 (1.12–1.39)
Never	2273	7180	1.17 (1.07–1.27)	1.24 (1.11–1.38)
Non-hair dye user	702	2614	1.0	1.0
*NA*	*533*	*2035*		
**Bleach**				
Ever	282	881	1.19 (1.04–1.37)	1.25 (1.06–1.48)
Never	4705	14752	1.18 (1.09–1.28)	1.27 (1.14–1.41)
Non-hair dye user	702	2614	1.0	1.0
*NA*	*878*	*3351*		
**Partial dye**				
Ever	2153	6686	1.20 (1.10–1.30)	1.28 (1.14–1.43)
Never	3247	10312	1.17 (1.07–1.27)	1.25 (1.12–1.39)
Non-hair dye user	702	2614	1.0	1.0
*NA*	*465*	*1986*		
***TOTAL***	***6567***	***21 598***		

*Odds ratios with 95% confidence interval are reported, multivariate model adjusted for birth year, parity, age at first birth, family history of breast cancer, menarche age, use of hormonal contraceptives, physical activity, alcohol use, body mass index and level of education.

^¥^Women reporting using hair dyes ‘Never’ or ‘1–2 times’ in their lifetime were classified as never-users, all other categories counting as ever-users.

^†^ Hair dye use according to the type of the dye was grouped into never and ever-users, answers ‘Rarely’ or ‘Never’ falling into category of never-users and ‘Often’ and ‘Quite often’ into ever-users. Non-users of hair dyes as defined in pooled ever vs. never-use were used as a reference category.

Regarding the different types of hair dyes, the odds ratios were increased with all types, when compared to non-hair dye users. The highest estimates were obtained for women who reported to have used temporary and/or semi-permanent dyes, with 32% and 31% increase in the odds of breast cancer (OR: 1.32, 95% CI: 1.16–1.52 and OR: 1.31, 95% CI: 1.17–1.46), respectively.

When including only invasive breast cancers in the analysis, a 21% increase in the estimated odds ratio was noted (OR: 1.21, 95% CI: 1.09–1.34, results not shown). In age group specific analysis, the odds ratio regarding ever vs. never use of hair dyes was the most increased in women born before 1950 (OR: 1.28, 95% CI: 1.10–1.48, Table 4). When stratifying the analysis by the reported menopausal status, increased odds ratio for ever-use of hair dyes was observed for post-menopausal women only (OR 1.25, 95% CI: 1.10–1.43 in contrast to OR 1.19, 95% CI: 0.81–1.75 for pre-menopausal women, results not shown). The attributable fraction of hair dyeing on breast cancer risk among the exposed was 18.7% (95% CI: 9.9%-26.5%) when estimated from the odds ratio of 1.23 (95% CI: 1.11–1.36). To reduce the risk of recall bias, we also did the analysis restricted to cases diagnosed more recently. We obtained an odds ratio of 1.30 (95% CI: 0.96–1.76) when including cases diagnosed in 2007 and OR 1.31 (95% CI: 1.07–1.61) when including diagnostic years 2006 and 2007.

**Table 4:** Results from the multivariate analysis of the association between breast cancer and hair dye use by birth year*.

	Born before 1950		Born 1950–1959		Born 1960 or after	
	N: Ca 2446, Co 7876		N: Ca 3004, Co 9994		N: Ca 1117, Co 3728	
	Cases/Controls	OR (CI 95%)	Cases/Controls	OR (CI 95%)	Cases/Controls	OR (CI 95%)
***Pooled ever-use***						
Ever	2044/6403	1.28 (1.10–1.48)	2709/8877	1.20 (1.02–1.40)	1040/3406	1.14 (0.85–1.54)
Never¥	359/1298	1.0	274/1013	1.0	69/303	1.0
*NA*	*43/175*		*21/104*		*8/19*	
***No*. *of dyes***						
0	227/790	1.0	148/561	1.0	37/159	1.0
1–2	132/508	0.96 (0.72–1.26)	126/452	1.25 (0.92–1.68)	32/144	0.89 (0.51–1.57)
3–9	352/1162	1.19 (0.96–1.48)	359/1157	1.27 (0.99–1.63)	118/418	0.97 (0.61–1.54)
10–34	751/2423	1.26 (1.03–1.53)	1016/3301	1.34 (1.07–1.68)	386/1265	1.10 (0.71–1.68)
35–89	553/1601	1.30(1.06–1.60)	804/2625	1.35 (1.07–1.70)	357/1079	1.15 (0.75–1.77)
≥ 90	388/1217	1.26 (1.01–1.56)	530/1794	1.34 (1.06–1.70)	179/644	0.96 (0.61–1.51)
*NA*	*43/175*		*21/104*		*8/19*	
***Age at first dye***						
<20	425/1464	1.03 (0.89–1.18)	659/2269	1.04 (0.91–1.19)	455/1479	1.09 (0.69–1.71)
20–29	644/1900	1.19 (1.05–1.36)	989/3101	1.12 (0.99–1.27)	428/1452	1.09 (0.70–1.70)
30–39	469/1405	1.17 (1.01–1.34)	656/2252	0.97 (0.85–1.11)	157/514	1.07 (0.67–1.70)
≥40	646/2176	1.0	534/1734	1.0	31/107	1.0
*NA*	*262/931*		*166/638*		*46/176*	
***Pooled age***						
<30	1069/3364	1.05 (0.95–1.16)	1648/5370	1.11 (1.02–1.21)	883/2931	1.03 (0.85–1.25)
≥30	1115/3581	1.0	1190/3986	1.0	188/621	1.0
*NA*	*262/931*		*166/638*		*46/176*	
***Temporary dye*** †						
Ever	211/631	1.38 (1.12–1.70)	275/851	1.27 (1.04–1.56)	87/244	1.27 (0.87–1.85)
Never	1298/3871	1.32 (1.13–1.54)	1895/6044	1.24 (1.06–1.46)	881/2854	1.15 (0.85–1.55)
Non-hair dye user	359/1298	1.0	274/1013	1.0	69/303	1.0
*NA*	*578/2076*		*560/2086*		*80/327*	
***Semi-permanent dye***						
Ever	864/2520	1.39 (1.19–1.63)	1197/3798	1.25 (1.05–1.47)	463/1398	1.21 (0.89–1.65)
Never	884/2699	1.27 (1.08–1.49)	1224/3908	1.24 (1.05–1.46)	538/1822	1.11 (0.82–1.51)
Non-hair dye user;	359/1298	1.0	274/1013	1.0	69/303	1.0
*NA*	*339/1359*		*309/1275*		*47/205*	
***Permanent dye***						
Ever	933/2918	1.24 (1.06–1.46)	1456/4741	1.23 (1.05–1.45)	670/2110	1.18 (0.87–1.59)
Never	889/2651	1.33 (1.13–1.56)	1046/3329	1.19 (1.01–1.41)	338/1200	1.08 (0.79–1.47)
Non-hair dye user	359/1298	1.0	274/1013	1.0	69/303	1.0
*NA*	*265/1009*		*228/911*		*40/115*	
***Bleach***						
Ever	88/269	1.20 (0.90–1.61)	124/389	1.29 (1.00–1.67)	70/223	1.12 (0.75–1.67)
Never	1594/4805	1.32 (1.13–1.53)	2203/6989	1.24 (1.06–1.46)	908/2958	1.15 (0.86–1.56)
Non-hair dye user	359/1298	1.0	274/1013	1.0	69/303	1.0
*NA*	*405/1504*		*403/1603*		*70/244*	
***Partial dye***						
Ever	620/1887	1.28 (1.08–1.52)	1027/3267	1.23 (1.04–1.46)	506/1532	1.25 (0.92–1.69)
Never	1246/3750	1.32(1.13–1.54)	1493/4789	1.23 (1.05–1.45)	508/1773	1.04 (0.77–1.41)
Non-hair dye user	359/1298	1.0	274/1013	1.0	69/303	1.0
*NA*	*221/941*		*210/925*		*34/120*	

* Odds ratios with 95% confidence interval are reported, multivariate model adjusted for birth year, parity, age at first birth, family history of breast cancer, menarche age, use of hormonal contraceptives, physical activity, alcohol use, body mass index and level of education.

^¥^Women reporting using hair dyes ‘Never’ or ‘1–2 times’ in their lifetime were classified as never-users, all other categories counting as ever-users.

^†^ Hair dye use according to the type of the dye was grouped into never and ever-users, answers ‘Rarely’ or ‘Never’ falling into category of never-users and ‘Often’ and ‘Quite often’ into ever-users. Non-users of hair dyes as defined in pooled ever-use were used as a reference category.

The bias-adjusted odds ratios and the parameters used as priors in the sensitivity analysis are presented in S1 Appendix. When adjusted for potential differential misclassification of hair dye use as an exposure, simulated bias-adjusted OR of 2.50 was observed, indicating 54% downward bias. Regarding bias due to non-response with respect to hair dye use, an adjusted OR of 1.04 was observed, suggesting 11% upward bias. With respect to uncontrolled confounding, a bias-adjusted OR of 1.46 (-21% bias) was estimated when the level of education was used as a surrogate to assess the role of socio-economic status.

Users of hair dye had a significant, 23% increased odds of breast cancer compared to non-users. The highest association was observed in women born before 1950 (28% increase in the odds). Furthermore, a substantial amount (19%) of the new breast cancer cases in women 60 years of age or less can potentially be attributable to the use of hair dye products.

The estimates did not significantly vary between different types of dyes. Most interestingly, temporary and semi-permanent dyes seemed to have greatest impact in the odds of breast cancer. Permanent hair colours containing higher concentrations of intermediates and oxidative agents have previously been thought to possess the most potentially hazardous effects. Nevertheless, many of the modern semi-permanent dyes contain a peroxidising agent and may thus be considered to act as permanent dyes with lower oxidative agent concentrations. Also, semi-permanent colours may contain potentially carcinogenic substances, as many of them contain an azo bond that after cleavage may result into the release of aromatic amines [16]. Even though Zheng et al. did not find an association between overall hair dye use and breast cancer, the risk estimates for the use of semi-permanent dyes were consistently above 1 and also higher compared to the estimates for the use of permanent hair dyes [17].

In a pooled analysis comparing ever and never-users of hair dyes, the odds ratio was the highest in women born before 1950. This appears logical as they have had a possibility to cumulate more hair dyeing episodes than their younger peers, for whom we might not yet see the possible effect of hair dye use in breast cancer incidence. Odds ratios for cumulative hair dye use, however, were the highest among women born between 1950 and 1959. If there is a causal association between hair dye use and breast cancer and the length of the induction period is assumed to be some 20–25 years, with our design, we are more likely to catch cases in women of that age group. Cases born earlier or later than this, whose disease might have been influenced by hair dye use, have already got their diagnosis before our ascertainment period 2000–2007, whereas the youngest cohort is still at risk. P-value from the likelihood test on the heterogeneity between odds ratios for different age groups (*P* = 0.024) also suggests that exposure over a long period of time carries a greater risk than short exposure.

Previous research on the association between hair dye use and risk of breast cancer has concluded with somewhat mixed results. While the research groups of Nasca [18], Cook [19] and Petro-Nustas [20] reported increased breast cancer risks in hair dye users, some studies have ended up with no association [16, 21–23]. A meta-analysis on the association between hair dye use and cancer risk concluded with a relative risk of 1.1 (95% CI: 0.9–1.2) for breast cancer, the total number of study subjects, however, remaining fewer than in the present study [24]. Support to the results presented here is provided by the Nordic Occupational Cancer Study (NOCCA), where the standardized incidence ratio obtained for breast cancer was 1.06 (1.01–1.10) among a Nordic cohort of female hairdressers (N = 1983) [25], which naturally mainly consisted of a somewhat similar study population, with respect to ethnicity.

As a retrospective study based on a self-administered questionnaire, our study is susceptible to differential recall bias. The respondents might have found it difficult to estimate the total number of hair dyes during their lifetime or to evaluate the types of dyes they have used and breast cancer cases might also have over-estimated their hair dye use if they have had a presumption of its association with their disease. We see, however, no reason to expect much difference between cases and controls in reporting ever vs. never-use of hair dyes as hair coloring is not commonly recognized as hazardous in regards to breast cancer. A reliability study by Shore at al. estimated self-reported hair dye information to be of good quality, obtaining a correlation coefficient (r) 0.86 for duration of hair dye use from two telephone interviews one year apart and the coefficients for cases and controls being almost similar [26].

It is still evident that we miss out cases with most aggressive types of cancer due to retrospective design. However, given the relatively similar diagnostic age distribution and the high overall user prevalence of hair dyes, it is unlikely that missing of these cases would largely bias the results. Also, results from the analysis including only cases from the last diagnostic years suggest that recall bias is does not play a major role in the estimations. Concerning the potential role of national population based mammography screening, while 89% of the cases diagnosed before the age of 60 years had had their first mammogram before the diagnose, some 87% of the controls aged < 60 years at questionnaire had done so, suggesting no differences between cases and controls in terms of screening attendance.

For further data-validation, we compared the distributions of age and education in the study population to the figures obtained from the official statistics representing the general population [27,28]. Questionnaire respondents were more often highly educated and older than women in the general population. When 26% of women in Finland between the ages 22 and 64 years were 55 years of age or older, the corresponding percentage in the study population was 43%. The difference in education was largest among the youngest study participants, with 16 percentage points more academically educated controls in the questionnaire, compared to that of the population. Stratifying the analysis by age, adjusting for education and estimating bias-adjusted odd ratios were one of the means in an effort to minimize the role of bias in the obtained results. Some uncertainty, however, remains whether e.g. the inability to observe association in the youngest age group is due bias or shorter period of time in possible disease latency.

To address for the role of recall bias, we estimated the level of differential misclassification of the exposure. Even though we assumed there not to be major differences between cases and controls in reporting whether or not they have ever used hair dyes, it is possible that more unexposed cases have falsely classified themselves as exposed if they believed there to be a relationship between hair dye use and breast cancer. Thus in the sensitivity analysis, the sensitivity was set to 90% for both cases and controls, whereas specificity among the cases was set to 80% and to 90% regarding controls. The observed bias-adjusted odds ratio of 2.50 and 54% downward bias imply that the results obtained in this study are highly sensitive to differential misclassification of the exposure. As stated, however, recall bias is not expected to have major impact in reporting ever vs. never use of hair dyes, whereas the situation regarding the number of dye episodes and different dye types is likely to be different and the level of uncertainty could be higher.

The response rates in the questionnaire were 69% in cases and 55% among controls. Based on this, we set the response probabilities with respect to hair dye exposure status in the sensitivity analysis to be 0.8 as for the exposed cases and 0.6 regarding non-exposed cases and correspondingly 0.6 as of the exposed controls and 0.5 as of the non-exposed controls. The probability was assumed to vary more among the cases whereas controls were likely to be more homogenous in responding hair dye-related questions, regardless of their exposure status. The estimated adjusted OR (1.04) implies that even if the response activity between the cases and controls and between the exposed and unexposed was assumed to be rather heterogeneous, the direction or the magnitude of the observed association does not majorly change.

As for uncontrolled confounding, socio-economic status was considered to be the most probable source of such bias in our questionnaire. According to official population statistics [28], on average 12% of the Finnish female population aged 25–65 years are academically educated and this figure was used as a prior when estimating the role of possible bias due to uncontrolled confounding. In the sensitivity analysis, the relative risk between high education (>16 years) and breast cancer was set to 1.36, as observed by Braaten et al. in 2004 [29]. The result of a bias-adjusted odds ratio of 1.46 with regards to uncontrolled confounding suggests that if the percentage of academically educated women in the study population would match the one of the general population—and given that education reliably serves as a proxy for socio-economic status, the obtained odds ratio would have been 21% higher.

There has been evidence on current commercial hair dyes containing 4-ABP and contaminated PPD has been speculated as the source of it. In 2003, Turesky et al. [9] found adducts of 4-ABP in 8 out of 11 hair dye samples, whereas Akyüz & Ata detected similar contamination in 28 out of 54 samples of different commercial hair dyes and in 11 out of 25 henna samples [10]. These evidences are further validated by the study of Amborosone and colleagues, where elevated levels of 4-ABP-DNA adducts were detected in the breast epithelial cells of hair dye users [30]. Also an IARC working group on the assessment of 4-aminobiphenyl suggested that frequent detection of 4-ABP adducts in non-smokers implies that there might be other environmental sources of exposure, contaminated hair dye products potentially being one of them [6]. Smoking was left out from our analysis as the step-wise model search ranked it as a non-significant variable. The observed odds ratios did not change when including smoking in the multivariate analysis, OR for ever-users of hair dye then being 1.22 (95% CI: 1.11–1.36, results not shown).

## Conclusions

Risk assessment in cosmetic industry is still largely self-regulated and lacks independent evaluation. Safety reports are often provided by the stakeholders of the cosmetic industry [31]. Considering the popularity of hair coloring in the modern societies, the results obtained in our study are worrying. Today, hair dyeing is an acceptable and essential part of beauty care and do-it-yourself dyes are affordable and easy to use. Focus should be targeted on the safety of commercial hair dyes and chemical substances they contain. Independent regulation and research on a governmental and organizational level should be strengthened and manufacturers of hair dye products ought to be provided with detailed guidelines and restrictions for development and manufacture.

Our results suggest that increasingly popular use of hair coloring products may be substantial in the etiology of new breast cancer cases. The study subjects, being permanent residents of Finland, were likely to be mostly of Caucasian race. Considering the fairly blonde and homogenous Finnish population, the results presented here may only be generalised to other western societies with Caucasian majorities. Hair coloring habits and products in terms of dye types, colors, and dye frequency are likely to differ between the cultures and ethnicities.

Even if the excess relative risk of breast cancer due to hair dye use is likely to be small at the individual-level, taken the prevalence of the exposure into account, its impact on public health can be considerable. We, however, acknowledge the limitations in retrospective study design and further research with prospective design is warranted before making conclusive arguments on the risks of hair dye use.

## Supporting Information

S1 Funding Statement (DOCX)

S1 STROBE Checklist (DOCX)

S1 Supporting Information Survey answering sheet (In Finnish). (PDF)

S2 Supporting Information Survey translation (In English). (PDF)

S1 Appendix (DOCX)
