# Supplementary material for: Does Hair Dye Use Increase the Risk of Breast Cancer? A Population-Based Case-Control Study of Finnish Women
Source: PLoS One. 2015 Aug 11;10(8):e0135190. doi: 10.1371/journal.pone.0135190 (PMC4532449; doi:10.1371/journal.pone.0135190)
Supplement: S1 Funding Statement — (DOCX) [file pone.0135190.s001.docx]

S1 Funding Statement

Funding statement for original research:

**Does Hair Dye Use Increase the Risk of Breast Cancer? A population-based case-control study of Finnish women**

*by Heikkinen et al.*

The original data collection was funded by the ZEG Berlin, Center for Epidemiology and Health Research and it produced a study “Levonorgestrel-releasing and copper intrauterine devices and the risk of breast cancer” by Dinger, Bardenheuer and Do Minh, published in 2010.

Sanna Heikkinen was supported by the Cancer Society of Finland for the present study (Epidemiological Researcher-grant), <http://www.cancer.fi/en/> . The funders had no role in study design, data collection and analysis, decision to publish, or preparation of the manuscript.
