# Supplementary material for: Does Hair Dye Use Increase the Risk of Breast Cancer? A Population-Based Case-Control Study of Finnish Women
Source: PLoS One. 2015 Aug 11;10(8):e0135190. doi: 10.1371/journal.pone.0135190 (PMC4532449; doi:10.1371/journal.pone.0135190)
Supplement: S1 STROBE Checklist — (DOCX) [file pone.0135190.s002.docx]

S1 Checklist

STROBE Statement—checklist of items that should be included in reports of observational studies

|  | Item No. | Recommendation | Page  No. | Relevant text from manuscript |
| --- | --- | --- | --- | --- |
| **Title and abstract** | 1 | (*a*) Indicate the study’s design with a commonly used term in the title or the abstract | 1 | “Does Hair Dye Use Increase the Risk of Breast Cancer?  A population-based case-control study of Finnish women” |
|  |  | (*b*) Provide in the abstract an informative and balanced summary of what was done and what was found | 2 | “We investigated whether the use of hair dyes increases breast cancer risk in women. --- After adjusting for major risk factors, the relative risk of breast cancer increased by 23% among those who used hair dyes compared to those who did not.” |
| Introduction | | | |  |
| Background/rationale | 2 | Explain the scientific background and rationale for the investigation being reported | 3 | “It has been suggested that certain chemical compounds, especially aromatic amines that are often present in commercial hair dyes and bleaches, may play a role in the etiology of some human cancers. Research results have though been inconclusive. --- Considering the extensive use of hair dyes, even a small increase in risk may have an immense impact on public health.” |
| Objectives | 3 | State specific objectives, including any prespecified hypotheses | 3 | “The main objective was to determine whether the use of hair dyes independently increases the risk of breast cancer.” |
| Methods | | | |  |
| Study design | 4 | Present key elements of study design early in the paper | 4 | “The study design was a retrospective, frequency matched population-based case-control study with a survey conducted in 2009. “ |
| Setting | 5 | Describe the setting, locations, and relevant dates, including periods of recruitment, exposure, follow-up, and data collection | 4 | “Case ascertainment was done in December 2008 --- All women in Finland aged 22 to 60 years and diagnosed with first in-situ or invasive breast cancer between 1^st^ January 2000 and 31^st^ December 2007 were considered eligible ---. Age-matched controls were sampled from the central population register. --- The exposure of primary interest in this study was the use of hair dyes. The survey Women’s Health and Use of Hormones --- also served in this study as the source of exposure information” |
| Participants | 6 | (*a*) *Cohort study*—Give the eligibility criteria, and the sources and methods of selection of participants. Describe methods of follow-up  *Case-control study*—Give the eligibility criteria, and the sources and methods of case ascertainment and control selection. Give the rationale for the choice of cases and controls  *Cross-sectional study*—Give the eligibility criteria, and the sources and methods of selection of participants | 4 | “Case ascertainment was done in December 2008 from the population-based, nationwide cancer registry, covering close to 100% of solid tumors. All women in Finland aged 22 to 60 years and diagnosed with first in-situ or invasive breast cancer between 1^st^ January 2000 and 31^st^ December 2007 were considered eligible. --- Age-matched controls were sampled from the central population register.” |
|  |  | (*b*) *Cohort study*—For matched studies, give matching criteria and number of exposed and unexposed  *Case-control study*—For matched studies, give matching criteria and the number of controls per case | 4 | “For the purpose of this study, re-matching by birth year was conducted to match the cases and controls in an exact ratio of 1:4.” |
| Variables | 7 | Clearly define all outcomes, exposures, predictors, potential confounders, and effect modifiers. Give diagnostic criteria, if applicable | 4 | **Outcome**: “--- first in-situ or invasive breast cancer ---.” **Exposures**: “The exposure of primary interest in this study was the use of hair dyes.--- The respondents were asked to estimate the cumulative number of hair dye episodes during life, age at first use and the types of dyes used. “ **Confounders or effect modifiers**: “Potential confounding factors, including parity, age at first birth, family history of breast cancer, menarche age, use of hormonal contraceptives, physical activity, alcohol use, BMI and education were included in the multivariate adjusted model” |
| Data sources/ measurement | 8* | For each variable of interest, give sources of data and details of methods of assessment (measurement). Describe comparability of assessment methods if there is more than one group | 4 | “The survey was self-administered and identical for cases and controls.--- Regarding the total number of hair dye episodes during life, response options were categorized as: never, 1-2 times, 3-9 times, 10-34 times, 35-89 times or 90 times or more. In a pooled analysis, women reporting using hair dyes ‘Never’ or ‘1-2 times’ in their lifetime were classified as never-users, all other categories counting as ever-users. Age at first dye was categorized as: Under 20 years of age, 20-29 years, 30-39 years, and 40 years or older. The different dye types were defined as: ‘Temporary’ = a color that rinses off at first or few washes, ‘Semi-permanent’ = a color that rinses off after several washes, ‘Permanent’ = a color that does not wash off, ‘Bleach’ = the hair was bleached before coloring and ‘Partial’ = the hair was only partially dyed, e.g. highlighted. The frequency of dyeings in each of the type-specific categories was classified as Often, Quite often, Rarely and Never.” |
| Bias | 9 | Describe any efforts to address potential sources of bias | 5 | “Owing to comprehensive cancer information from the population based cancer registry in Finland, the coverage of cancer diagnoses was close to complete and the role of a possible selection/ascertainment bias was considered to be negligible. A deterministic sensitivity analysis was conducted in an effort to assess other potential sources of bias affecting the observed findings. Misclassification of the main exposure of interest (hair dye use), non-response bias with respect to hair dye use and a role of socio-economic status as an uncontrolled confounder were considered and bias-adjusted odds ratios with bias percentages are presented.” |
| Study size | 10 | Explain how the study size was arrived at | 4 | “**All** women in Finland aged 22 to 60 years and diagnosed with first in-situ or invasive breast cancer between 1^st^ January 2000 and 31^st^ December 2007 were considered eligible. Age-matched controls were sampled from the central population register.” |

Continued on next page

| Quantitative variables | 11 | Explain how quantitative variables were handled in the analyses. If applicable, describe which groupings were chosen and why | 7, 9,10 | *Categorizings used are presented in tables 2,3 and 4 and groupings made for analytical purposes are explained in the footnotes of the appropriate tables*. “ ¥Women reporting using hair dyes ‘Never’ or ‘1-2 times’ in their lifetime were classified as never-users, all other categories counting as ever-users. † Hair dye use according to the type of the dye was grouped into never and ever-users, answers ‘Rarely’ or ‘Never’ falling into category of never-users and ‘Often’ and ‘Quite often’ into ever-users. Non-users of hair dyes as defined in pooled ever vs. never-use were used as a reference category.” |
| --- | --- | --- | --- | --- |
| Statistical methods | 12 | (*a*) Describe all statistical methods, including those used to control for confounding | 4-5 | “We report odds ratios (OR), with their 95% confidence intervals (CI) from the conditional logistic regression model applied to frequency matched study design. Potential confounding factors --- were included in the multivariate adjusted model, as suggested by the step wise model search. --- Attributable fraction in the exposed --- was calculated with a formula presented by Greenland ((OR-1)/OR) x 100). --- The sensitivity analysis was performed with Stata version 12, using the Episensi-command” |
|  |  | (*b*) Describe any methods used to examine subgroups and interactions | 5 | “To identify differences in the risk of breast cancer in women with different type of hair dye exposure history, results stratified by birth year cohorts are presented.” |
|  |  | (*c*) Explain how missing data were addressed | 5 | “Subjects with missing values in any of the covariates in the fitted model were excluded.” |
|  |  | (*d*) *Cohort study*—If applicable, explain how loss to follow-up was addressed  *Case-control study*—If applicable, explain how matching of cases and controls was addressed  *Cross-sectional study*—If applicable, describe analytical methods taking account of sampling strategy | 4 | “--- re-matching by birth year was conducted to match the cases and controls --- We report odds ratios (OR), with their 95% confidence intervals (CI) from the conditional logistic regression model applied to frequency matched study design.” |
|  |  | (*e*) Describe any sensitivity analyses | 5 | “A deterministic sensitivity analysis was conducted in an effort to assess other potential sources of bias affecting the observed findings. Misclassification of the main exposure of interest (hair dye use), non-response bias with respect to hair dye use and a role of socio-economic status as an uncontrolled confounder were considered and bias-adjusted odds ratios with bias percentages are presented. --- The sensitivity analysis was performed with Stata version 12, using the Episensi-command, as introduced by Orsini and colleagues**.**” |
| Results | | | | |
| Participants | 13* | (a) Report numbers of individuals at each stage of study—eg numbers potentially eligible, examined for eligibility, confirmed eligible, included in the study, completing follow-up, and analysed | 5 | **Cases**: “Of the 14 815 breast cancer cases identified from the cancer registry, 1550 had died before the start of the study, leaving 13 265 cases in the sample. After re-matching the cases and controls by birth year, 10 448 women with breast cancer were left in the data. Of these, 951 were excluded due to any previous malignancy, leaving 9 537 cancer cases for the study, out of which 6567 responded to the survey (69%).” **Controls**: “As of the controls, 41 978 subjects remained in the sample after the re-matching, out of the 64 353 originally sampled. Of these 23 114 responded to the survey (55%). A previous malignancy was reported by 1516 controls and these were excluded, leaving 21 598 controls in the analytical data set. ” |
|  |  | (b) Give reasons for non-participation at each stage | 5 | **Cases**: “1550 had died before the start of the study --- 951 were excluded due to any previous malignancy” **Controls**: “A previous malignancy was reported by 1516 controls and these were excluded” |
|  |  | (c) Consider use of a flow diagram |  | *Detailed flow chart of the data formation is given in the validity assessment manuscript currently under review (submitted 1/2015).* |
| Descriptive data | 14* | (a) Give characteristics of study participants (eg demographic, clinical, social) and information on exposures and potential confounders | 5-6 | “With respect to morphology of the cancers, 5248 (80%) were ductal carcinomas, of which 4758 (91%) invasive and 1022 (16%) lobular carcinomas, of which 1002 (98%) invasive.--- Prevalence of other breast cancer risk factors according to hair dye use (24 479 users and 3 316 non-users) are shown in Table 2.” |
|  |  | (b) Indicate number of participants with missing data for each variable of interest | 7-10 | Tables 2, 3 and 4 |
|  |  | (c) *Cohort study*—Summarise follow-up time (eg, average and total amount) |  |  |
| Outcome data | 15* | *Cohort study*—Report numbers of outcome events or summary measures over time |  |  |
|  |  | *Case-control study—*Report numbers in each exposure category, or summary measures of exposure | 9 | Table 3 |
|  |  | *Cross-sectional study—*Report numbers of outcome events or summary measures |  |  |
| Main results | 16 | (*a*) Give unadjusted estimates and, if applicable, confounder-adjusted estimates and their precision (eg, 95% confidence interval). Make clear which confounders were adjusted for and why they were included | 4, 9 (Tab. 3), 10 (Tab. 4) | “We report odds ratios (OR), with their 95% confidence intervals ---. Potential confounding factors --- were included in the multivariate adjusted model, as suggested by the step wise model search.” Tables 3 and 4. |
|  |  | (*b*) Report category boundaries when continuous variables were categorized |  | *Given in each table or the appropriate footnote.* |
|  |  | (*c*) If relevant, consider translating estimates of relative risk into absolute risk for a meaningful time period |  |  |

Continued on next page

| Other analyses | 17 | Report other analyses done—eg analyses of subgroups and interactions, and sensitivity analyses | 4, 10 (Tab.4) 8, 20 (App.1) | “ --- results stratified by birth year cohorts are presented. *(Table 4)* --- The bias-adjusted odds ratios and the parameters used as priors in the sensitivity analysis are presented in Appendix 1.” |
| --- | --- | --- | --- | --- |
| Discussion | | | | |
| Key results | 18 | Summarise key results with reference to study objectives | 11 | “Users of hair dye had a significant 23% increased risk of breast cancer compared to non-users. The highest association was observed in women born before 1950 (28% increase in relative risk). Furthermore, a substantial amount (19%) of the new breast cancer cases in women 60 years of age or less can potentially be attributable to the use of hair dye products.  The risk estimates did not significantly vary between different types of dyes.” |
| Limitations | 19 | Discuss limitations of the study, taking into account sources of potential bias or imprecision. Discuss both direction and magnitude of any potential bias | 12-14 | “--- we miss out cases with most aggressive types of cancer due to retrospective design. --- As a retrospective study based on a self-administered survey, our study is susceptible to differential recall bias.--- recall bias is not expected to have major impact in reporting ever vs. never use of hair dyes --- The estimated adjusted OR (1.04) [*regarding non-response bias*] implies that even if the response activity between the cases and controls and between the exposed and unexposed was assumed to be rather heterogeneous, the direction or the magnitude of the observed risk effect does not majorly change. --- The result of a bias-adjusted odds ratio of 1.46 with regards to uncontrolled confounding suggests that if the percentage of academically educated women in the study population would match the one of the general population – and given that education reliably serves as a proxy for socio-economic status, the obtained odds ratio would have been 21% higher.” |
| Interpretation | 20 | Give a cautious overall interpretation of results considering objectives, limitations, multiplicity of analyses, results from similar studies, and other relevant evidence | 15 | “Our results suggest that increasingly popular use of hair coloring products may be substantial in the etiology of new breast cancer cases. --- Even if the excess risk of breast cancer due to hair dye use is likely to be small at the individual-level, taken the prevalence of the exposure into account, its impact on public health can be considerable. We, however, acknowledge the limitations in retrospective study design and further research with prospective design is warranted before making conclusive arguments on the risks of hair dye use.” |
| Generalisability | 21 | Discuss the generalisability (external validity) of the study results | 15 | “--- the results presented here may only be generalised to other western societies with Caucasian majorities. Hair coloring habits and products in terms of dye types, colors, and dye frequency are likely to differ between the cultures and ethnicities.” |
| Other information | |  | | |
| Funding | 22 | Give the source of funding and the role of the funders for the present study and, if applicable, for the original study on which the present article is based | 15 | “The original data collection was funded by the ZEG Berlin, Center for Epidemiology and Health Research and it produced a study “Levonorgestrel-releasing and copper intrauterine devices and the risk of breast cancer” by Dinger, Bardenheuer and Do Minh, published in 2010 [13]. Sanna Heikkinen was supported by the Cancer Society of Finland (Epidemiological Researcher-grant). Authors have no conflict of interests. “ |

*Give information separately for cases and controls in case-control studies and, if applicable, for exposed and unexposed groups in cohort and cross-sectional studies.

**Note:** An Explanation and Elaboration article discusses each checklist item and gives methodological background and published examples of transparent reporting. The STROBE checklist is best used in conjunction with this article (freely available on the Web sites of PLoS Medicine at http://www.plosmedicine.org/, Annals of Internal Medicine at http://www.annals.org/, and Epidemiology at http://www.epidem.com/). Information on the STROBE Initiative is available at www.strobe-statement.org.
