# Supplementary material for: Does Hair Dye Use Increase the Risk of Breast Cancer? A Population-Based Case-Control Study of Finnish Women
Source: PLoS One. 2015 Aug 11;10(8):e0135190. doi: 10.1371/journal.pone.0135190 (PMC4532449; doi:10.1371/journal.pone.0135190)
Supplement: S1 Supporting Information — (PDF) [file pone.0135190.s003.pdf]

HELSINGIN YLIOPISTO

Helsingin yliopiston Kansanterveystieteen laitoksen tutkimus:

# Naisten terveys ja hormonien käyttö

Tutkimusryhmämme selvittää HORMONIVALMISTEIDEN KÄYTÖN ja NAISTEN TERVEYDEN SUHDETTA. Tässä kyselylomakkeessa on pyritty kattavasti huomioimaan tärkeimmät rintasyövän riskitekijät sekä tekijöitä, joista ei ole riittävää tietoa.

Olet mukana Suomen väestöstä tehdyn neljänkymmenen tuhannen naisen otannan perusteella.

Jotta tutkimuksen tulokset olisivat luotettavia, on tärkeää, että kaikki lomakkeen saaneet vastaavat.

Voit jättää vastauksesi oheisessa palautuskuoressa ilman postimerkkiä postin kuljetettavaksi.

Kiitämme jo etukäteen siitä, että jaksat vastata kaikkiin kysymyksiin.

HUOMAA! Henkilöllisyytesi ja tietosi pidetään täysin salassa. Tutkimuksen tuloksia ei tarkastella henkilöittäin, vaan ne esitetään tilastollisina kokonaisuuksina taulukoiden muodossa.

# VASTAAMISOHJEET

Rastita kunkin kysymyksen / osakysymyksen kohdalla vain yksi vaihtoehto, paitsi kysymyksissa 32 ja 42, joissa voi rastittaa useampia vaihtoehtoja.

Käytä kuulakärkikynää, koska tämä kysely tallennetaan optisella laitteella.

**Vastausmalli 1:**

**Kuinka usein syöt seuraavia ruokia?**

| | Päivittäin | Noin 3-6 päivänä viikossa | Noin 4-10 päivänä kuukaudessa | Noin 1-2 päivänä kuukaudessa | Harvemmin tai ei koskaan |
|---|---|---|---|---|---|
| Ruisleipää | ☒ | ☐ | ☐ | ☐ | ☐ |
| Tuoreita hedelmiä | ☒ | ☐ | ☐ | ☐ | ☐ |
| Lohta tai kirjolohta | ☐ | ☐ | ☐ | ☐ | ☒ |

**Rastit näin, kun syöt päivittäin ruisleipää ja tuoreita vihanneksia, mutta et lainkaan lohikalaa.**

**Vastausmalli 2:**

**Kuinka paljon painat?**
**merkitse ruutuun sadat kilot, kymmenkilot ja kilot**

| | 0 | 1 | 2 | 3 | 4 | 5 | 6 | 7 | 8 | 9 |
|---|---|---|---|---|---|---|---|---|---|---|
| sadat kilot | ☒ | ☐ | ☐ | | | | | | | |
| kymmenet kilot | ☐ | ☐ | ☐ | ☐ | ☐ | ☐ | ☐ | ☒ | ☐ | ☐ |
| kilot | ☐ | ☐ | ☒ | ☐ | ☐ | ☐ | ☐ | ☐ | ☐ | ☐ |

**Merkitset näin, jos painat 72 kiloa**

**Vastausmalli 3:**

**Mitä vitamiineja käytät tällä hetkellä?**

C-vitamiinia ja D-vitamiinia

**Näin kirjoitat, jos käytät tällä hetkellä vain C- ja D-vitamiinia.**



7594352252

## TÄSTÄ ALKAVAT VARSINAISET KYSYMYKSET

### 1. Oletko ollut koskaan raskaana?

- ☐ kyllä
- ☐ en koskaan, siirry kysymykseen 7.

### 2. Kuinka monta lasta olet synnyttänyt?

- ☐ yhden
- ☐ kaksi
- ☐ kolme - neljä
- ☐ viisi - kuusi
- ☐ seitsemän tai enemmän

### 3. Minkä ikäisenä synnytit ensimmäisen lapsesi? Olin...

- ☐ 15 vuotias tai nuorempi
- ☐ 16-20-vuotias
- ☐ 21-25-vuotias
- ☐ 26-30-vuotias
- ☐ 31-35-vuotias
- ☐ 36-40-vuotias
- ☐ 41-vuotias tai vanhempi

### 4. Kuinka monta keskenmenoa tai aborttia sinulla on ollut?

- ☐ ei yhtään
- ☐ yksi
- ☐ kaksi - kolme
- ☐ neljä tai useampi

### 5. Oletko koskaan imettänyt?

- ☐ kyllä
- ☐ en, siirry kysymykseen 7.

**6. Kuinka pitkän ajan olet imettänyt yhteensä, kun kaikki lapsesi otetaan huomioon?**

- ☐ en ollenkaan
- ☐ viikon
- ☐ kaksi - kolme viikkoa
- ☐ kuukauden
- ☐ kaksi - kolme kuukautta
- ☐ neljä - viisi kuukautta
- ☐ puoli vuotta - vuoden
- ☐ yli vuoden, mutta alle kaksi vuotta
- ☐ kaksi vuotta tai enemmän

**7. Oletko koskaan käyttänyt raskauden ehkäisyyn ehkäisypillereitä, ehkäisylaastareita tai pistoksina tai ihonalaisena kapselina annettavaa hormoniehkäisyä?**

- ☐ kyllä
- ☐ en, siirry kysymykseen 10.

**8. Minkä ikäisenä aloitit käyttää raskauden ehkäisyyn ehkäisypillereitä, ehkäisylaastareita tai pistoksina tai ihonalaisena kapselina annettavaa hormoniehkäisyä? Olin..**

- ☐ 15 vuotias tai nuorempi
- ☐ 16-20-vuotias
- ☐ 21-25-vuotias
- ☐ 26-30-vuotias
- ☐ 31-35-vuotias
- ☐ 36-40-vuotias
- ☐ 41-vuotias tai vanhempi
- ☐ en osaa sanoa

**9. Käytätkö nyt raskauden ehkäisyyn ehkäisypillereitä, ehkäisylaastareita tai pistoksina tai ihonalaisena kapselina annettavaa hormoniehkäisyä?**

- ☐ kyllä
- ☐ en

**10. Oletko koskaan käyttänyt hormonivalmisteita puuttuvien, kivuliaiden tai epäsäännöllisten kuukautisten hoitoon tai aknen (finnien) hoitoon?**

- ☐ kyllä
- ☐ en, siirry kysymykseen 12.

**11. Minkä ikäisenä aloit käyttää hormonivalmisteita puuttuvien, kivuliaiden tai epäsäännöllisten kuukautisten hoitoon tai aknen (finnien) hoitoon? Olin..**

☐ 15 vuotias tai nuorempi

☐ 16-20-vuotias

☐ 21-25-vuotias

☐ 26-30-vuotias

☐ 31-35-vuotias

☐ 36-40-vuotias

☐ 41-vuotias tai vanhempi

☐ en osaa sanoa

**12. Kuinka pitkän ajan yhteensä olet käyttänyt ehkäisypillereitä, ehkäisylaastareita tai pistoksena tai ihonalaisena kapselina annettavaa hormonivalmisteita ehkäisyyn, puuttuvien, kivuliaiden tai epäsäännöllisten kuukautisten hoitoon tai aknen (finnien) hoitoon?**
**(yritä arvioida käytön yhteenlaskettua aikaa)**

☐ En ole koskaan käyttänyt, siirry kysymykseen 15.

☐ alle kuukauden

☐ kuukauden - puolen vuoden ajan

☐ yli puoli vuotta, mutta alle kaksi vuotta

☐ kaksi - neljä vuotta

☐ yli neljä vuotta - kahdeksan vuotta

☐ yli kahdeksan vuotta - kaksitoista vuotta

☐ yli kaksitoista vuotta - kuusitoista vuotta

☐ yli kuusitoista vuotta - kaksikymmentä vuotta

☐ yli kaksikymmentä vuotta - kaksikymmentäviisi vuotta

☐ yli kaksikymmentäviisi vuotta

**13. Kuinka vanhana lopetit kaikki ehkäisypillereiden, ehkäisylaastareiden tai pistoksina tai ihonalaisena kapselina annettavien hormonivalmisteiden käytön? Huomioi vastauksessasi ehkäisy, puuttuvien tai epäsäännöllisten kuukautisten hoito tai aknen (finnien) hoito.**

☐ en ole vielä lopettanut

☐ 15 vuotias tai nuorempi

☐ 16-20-vuotias

☐ 21-25-vuotias

☐ 26-30-vuotias

☐ 31-35-vuotias

☐ 36-40-vuotias

☐ 41-vuotias tai vanhempi

**14. Voitko yrittää palauttaa mieleen, mitä ehkäisypillereitä, ehkäisylaastareita tai pistoksina tai ihonalaisena kapselina annettavia hormonivalmisteita olet käyttänyt?**
**Merkitse seuraavaan taulukkoon valmisteen nimi, käytön aloittamisvuosi ja lopettamisvuosi sekä syy lopettamiseen (esim sivuvaikutus, haluttu raskaus).**

| Valmisteen nimi | käytön alkuvuosi | kuukausi | käytön lopettamisvuosi | kuukausi | lopettamisen syy |
|---|---|---|---|---|---|
| | | | | | |
| | | | | | |
| | | | | | |
| | | | | | |
| | | | | | |

**15. Oletko koskaan käyttänyt hormonikierukkaa (esim Levonova, Mirena) tai ehkäisykierukkaa (esim kuparikierukkaa) ?** Jos et ole käyttänyt koskaan mitään ehkäisykierukkaa, siirry kysymykseen 17.

**a. kuparikierukkaa**

☐ kyllä

☐ en

☐ en osaa sanoa

**b. hormonikierukkaa**

☐ kyllä

☐ en

☐ en osaa sanoa

**c. muuta ehkäisykierukkaa**

☐ kyllä

☐ en

☐ en osaa sanoa

**16. Voitko yrittää palauttaa mieleen, mitä ehkäisykierukkaa olet käyttänyt?**
**Merkitse seuraavaan taulukkoon valmisteen nimi, käytön aloittamisvuosi ja lopettamisvuosi.**

| Kierukan nimi | käytön alkuvuosi | kuukausi | käytön lopettamisvuosi | kuukausi | käytön lopettamisen syy (esim. raskaus) |
|---|---|---|---|---|---|
| | | | | | |
| | | | | | |
| | | | | | |
| | | | | | |
| | | | | | |

**17. Minkä ikäinen olit, kun sinulla oli ensimmäiset kuukautiset?**

- ☐ minulla ei ole koskaan ollut kuukautisia
- ☐ 12-vuotias tai nuorempi
- ☐ 13-vuotias
- ☐ 14-vuotias
- ☐ 15-vuotias
- ☐ 16-vuotias
- ☐ 17-vuotias
- ☐ 18-vuotias
- ☐ 19-vuotias
- ☐ 20-vuotias
- ☐ 21-vuotias
- ☐ 22-vuotias tai vanhempi
- ☐ en osaa sanoa

**18. Onko sinulla edelleen kuukautiset?**

- ☐ ei, koska olen raskaana
- ☐ ei, koska kohtuni on poistettu
- ☐ kyllä, säännölliset
- ☐ kyllä, epäsäännölliset
- ☐ en ole varma, koska ne ovat epäsäännölliset
- ☐ kuukautiseni ovat palanneet hormonihoidon takia
- ☐ ei, kuukautiseni ovat pysyvästi loppuneet luonnollisella tavalla.

Kuinka vanha olit silloin? ☐☐

**19. Oletko saanut vaihdevuosien tai munasarjojen poiston vuoksi hormonikorvaushoitoa?**

- ☐ kyllä
- ☐ en, siirry kysymykseen 25.
- ☐ en osaa sanoa

**20. Kuinka vanha olit, kun hormonikorvaushoito alkoi?**

- ☐ 40-vuotias tai nuorempi
- ☐ 41-45-vuotias
- ☐ 46-50-vuotias
- ☐ 51-55-vuotias
- ☐ 56-60-vuotias
- ☐ 61-65-vuotias
- ☐ 66-vuotias tai vanhempi
- ☐ en osaa sanoa

**21. Käytätkö tällä hetkellä hormonikorvaushoitoa?**

- ☐ kyllä
- ☐ en

**22. Kuinka vanhana lopetit hormonikorvaushoidon?**

- ☐ 40-vuotias tai nuorempi
- ☐ 41-45-vuotias
- ☐ 46-50-vuotias
- ☐ 51-55-vuotias
- ☐ 56-60-vuotias
- ☐ 61-65-vuotias
- ☐ 66-70-vuotias
- ☐ 71-75-vuotias
- ☐ 76-vuotias tai vanhempi

**23. Kauanko olet käyttänyt hormonikorvaushoitoa yhteensä?**
**(yritä arvioida käytön yhteenlaskettua aikaa)**

- ☐ alle kuukauden
- ☐ kuukauden - puolen vuoden ajan
- ☐ yli puoli vuotta, mutta alle kaksi vuotta
- ☐ kaksi - neljä vuotta
- ☐ yli neljä vuotta - kahdeksan vuotta
- ☐ yli kahdeksan vuotta - kaksitoista vuotta
- ☐ yli kaksitoista vuotta - kuusitoista vuotta
- ☐ yli kuusitoista vuotta - kaksikymmentä vuotta
- ☐ yli kaksikymmentä vuotta - kaksikymmentäviisi vuotta
- ☐ yli kaksikymmentäviisi vuotta

**24. Voitko yrittää palauttaa mieleen, mitä hormonikorvaushoitoa olet saanut?**
**Merkitse seuraavaan taulukkoon valmisteen nimi, käytön aloittamisvuosi ja lopettamisvuosi sekä syy lopettamiseen (esim sivuvaikutus)**

| Valmisteen nimi | käytön alkuvuosi | käytön lopettamisvuosi | lopettamisen syy |
|---|---|---|---|
| | | | |
| | | | |
| | | | |
| | | | |
| | | | |

**25. Oletko koskaan tutkinut, onko rinnoissasi kyhmyjä**

- ☐ en, siirry kysymykseen 27.
- ☐ kyllä

**26. Kuinka usein tutkit, onko rinnoissasi kyhmyjä?**

- ☐ useammin kuin kaksi kertaa vuodessa
- ☐ kaksi kertaa vuodessa
- ☐ kerran vuodessa
- ☐ harvemmin kuin kerran vuodessa

**27. Oletko koskaan ollut mammografia-tutkimuksessa (rintojen röntgentutkimuksessa)?**

- ☐ kyllä
- ☐ en, siirry kysymykseen 31.

**28. Kuinka vanhana kävit ensimmäistä kertaa mammografiatutkimuksessa?**

- ☐ alle 40-vuotiaana
- ☐ 40-44-vuotiaana
- ☐ 45-49-vuotiaana
- ☐ 50-54-vuotiaana
- ☐ 55-59-vuotiaana
- ☐ 60-64-vuotiaana
- ☐ 65-69-vuotiaana
- ☐ 70-vuotiaana tai vanhempana

**29. Kuinka vanha olit, kun kävit viimeksi mammografiatutkimuksessa?**

- ☐ alle 40-vuotiaana
- ☐ 40-44-vuotiaana
- ☐ 45-49-vuotiaana
- ☐ 50-54-vuotiaana
- ☐ 55-59-vuotiaana
- ☐ 60-64-vuotiaana
- ☐ 65-69-vuotiaana
- ☐ 70-vuotiaana tai vanhempana

**30. Kuinka monta kertaa yhteensä olet käynyt mammografiatutkimuksessa?**

- ☐ kerran
- ☐ kaksi - kolme kertaa
- ☐ neljä - viisi kertaa
- ☐ kuusi kertaa tai useammin

**31. Onko sinulla koskaan todettu hyvänlaatuista kyhmyä rinnassa?**

- ☐ kyllä
- ☐ ei, siirry kysymykseen 33.
- ☐ en osaa sanoa, siirry kysymykseen 33.

**32. Millainen hyvänlaatuinen kyhmy oli tai kyhmyt olivat? Merkitse myös arviosi toteamisvuodesta.**
**Jos sinulla on todettu useita kertoja samanlaatuinen kyhmy, merkitse vain ensimmäinen toteamisvuosi.**

| | toteamisvuosi |
|---|---|
| ☐ en tiedä minkälainen kyhmy | ☐☐☐☐ |
| ☐ yksittäinen kysta | ☐☐☐☐ |
| ☐ hyperplasia | ☐☐☐☐ |
| ☐ mastiitti | ☐☐☐☐ |
| ☐ hormonikysta | ☐☐☐☐ |
| ☐ adenoma | ☐☐☐☐ |
| ☐ kystoadenoma | ☐☐☐☐ |
| ☐ fibroadenoma | ☐☐☐☐ |
| ☐ jokin muu | ☐☐☐☐ |

**33. Onko sinulla koskaan todettu rintasyöpää?**

☐ ei, siirry kysymykseen 38.

☐ en osaa sanoa, siirry kysymykseen 38.

☐ kyllä

**34. Minä vuonna rintasyöpä todettiin?** Vuosi ☐☐☐☐

**35. Todettiinko silloin etäpesäkkeitä?**

☐ ei

☐ en osaa sanoa

☐ kyllä

**36. Miten rintasyöpä hoidettiin?**

**a. leikkauksella**

☐ kyllä

☐ ei

**b. solumyrkyillä (kemoterapialla)**

☐ kyllä

☐ ei

**c. sädehoidolla**

☐ kyllä

☐ ei



3418352253

**37. Mitä muuta hoitoa rintasyöpään annettiin?**______________________________

**38. Onko sinulla todettu jokin muu syöpä?**

- ☐ ei, siirry kysymykseen 41.
- ☐ en osaa sanoa, siirry kysymykseen 41.
- ☐ kyllä

**39. Mikä syöpä se oli?** ______________________________

**40. Minä vuonna se todettiin?** Vuosi ☐☐☐☐

**41. Onko äidilläsi, siskollasi tai tyttärelläsi todettu rintasyöpä?**

- ☐ ei, siirry kysymykseen 43.
- ☐ kyllä

**42. Kellä heistä on todettu rintasyöpä?**

- ☐ äidillä
- ☐ siskolla
- ☐ tyttärellä, minulla on ☐☐ siskoa ja ☐☐ tytärtä

**43. Mikä on siviilisäätysi**

- ☐ naimaton
- ☐ naimisissa tai avoliitossa
- ☐ eronnut tai asumuserossa
- ☐ leski

**44. Mikä on peruskoulutuksesi? (valitse vain yksi vaihtoehto)**

- ☐ ei peruskoulutusta
- ☐ kansakoulu/kansalaiskoulu
- ☐ peruskoulu/keskikoulu
- ☐ ylioppilastutkinto
- ☐ ammattikorkeakoulu tai vastaava
- ☐ korkeakoulututkinto

**45. Kuinka pitkä olet?**
**merkitse ruutuun metri, 10-sentit ja sentit**

| | 0 | 1 | 2 | 3 | 4 | 5 | 6 | 7 | 8 | 9 |
|---|---|---|---|---|---|---|---|---|---|---|
| metrit | ☐ | ☐ | ☐ | | | | | | | |
| 10-sentit | ☐ | ☐ | ☐ | ☐ | ☐ | ☐ | ☐ | ☐ | ☐ | ☐ |
| sentit | ☐ | ☐ | ☐ | ☐ | ☐ | ☐ | ☐ | ☐ | ☐ | ☐ |

**46. Kuinka paljon painat?**
**merkitse ruutuun sadat kilot, kymmenkilot ja kilot**

| | 0 | 1 | 2 | 3 | 4 | 5 | 6 | 7 | 8 | 9 |
|---|---|---|---|---|---|---|---|---|---|---|
| sadat kilot | ☐ | ☐ | ☐ | | | | | | | |
| 10-kilot | ☐ | ☐ | ☐ | ☐ | ☐ | ☐ | ☐ | ☐ | ☐ | ☐ |
| kilot | ☐ | ☐ | ☐ | ☐ | ☐ | ☐ | ☐ | ☐ | ☐ | ☐ |

**47. Oletko koskaan polttanut savukkeita säännöllisesti (päivittäin tai lähes päivittäin)?**

☐ en, siirry kysymykseen 53.

☐ kyllä

**48. Minkä ikäisenä aloitit savukkeiden polton säännöllisesti?**

☐ alle 13 vuotiaana

☐ 13-15-vuotiaana

☐ 16-18-vuotiaana

☐ 19-21-vuotiaana

☐ 22-24-vuotiaana

☐ 25-27-vuotiaana

☐ 28-vuotiaana tai vanhempana

**49. Montako vuotta olet yhteensä polttanut savukkeita? Yritä vähentää määrästä tupakkalakot.**

☐ alle vuoden

☐ 1-2 vuotta

☐ 3-7 vuotta

☐ 8-12 vuotta

☐ 13-19-vuotta

☐ 20 vuotta tai kauemmin

**50. Mikä on ollut keskimäärin poltettujen savukkeiden määrä päivässä?**

☐ alle 5 savuketta

☐ 5-9 savuketta

☐ 10-19 savuketta

☐ 20-29 savuketta

☐ 30 savuketta tai enemmän



2457352255

**51. Poltatko edelleen savukkeita?**

- ☐ kyllä, siirry kysymykseen 53.
- ☐ en

**52. Minkä ikäisenä lopetit savukkeiden polton?**

- ☐ alle 15 vuotiaana
- ☐ 16-20-vuotiaana
- ☐ 21-30-vuotiaana
- ☐ 31-40-vuotiaana
- ☐ 41-50-vuotiaana
- ☐ 51-60-vuotiaana
- ☐ 61-vuotiaana tai vanhempana

**53. Kuinka usein juot alkoholijuomia?**

- ☐ en koskaan
- ☐ vain erityisissä tilaisuuksissa
- ☐ kolmena päivänä viikossa tai harvemmin
- ☐ neljänä päivänä viikossa tai useammin

**54. Kuinka usein ruumiillinen liikunta aiheuttaa sinulle selvän hengästymisen ja pulssin lisääntymisen?**

- ☐ ei koskaan
- ☐ kerran kuussa tai harvemmin
- ☐ 2-3 kertaa kuussa
- ☐ noin kerran viikossa
- ☐ useita kertoja viikossa
- ☐ päivittäin

**55. Onko sinulla koskaan ollut jokin muu lääkitys kuin hormonivalmiste, jota olet käyttänyt yli kuukauden ajan?**

- ☐ ei
- ☐ kyllä, mikä valmiste? ________________________________________

________________________________________

**56. Kun ajattelet aikaa kymmenen vuotta sitten, arvioi, montako tuntia nukuit yleensä vuorokaudessa?**

- ☐ 6 tuntia tai vähemmän
- ☐ 6.5 tuntia
- ☐ 7 tuntia
- ☐ 7.5 tuntia
- ☐ 8 tuntia
- ☐ 8.5 tuntia
- ☐ 9 tuntia
- ☐ 9.5 tuntia
- ☐ 10 tuntia tai enemmän

9813352258

**57. Oletko koskaan värjäyttänyt tai värjännyt hiuksiasi?**

- ☐ en koskaan, siirry kysymykseen 60.
- ☐ 1-2 kertaa
- ☐ 3-9 kertaa
- ☐ 10-34 kertaa
- ☐ 35-89 kertaa
- ☐ 90 kertaa tai useammin

**58. Minkä ikäisenä värjäytit tai värjäsit hiuksesi ensimmäisen kerran?**

- ☐ alle 20 vuotiaana
- ☐ 20-29 vuotiaana
- ☐ 30-39 vuotiaana
- ☐ 40-vuotiaana tai vanhempana

**59. Koeta muistella, millä tavalla hiustesi värjäykset ovat tapahtuneet.**

**a.värillä, joka huuhtoutuu seuraavassa hiusten pesussa.**

- ☐ usein
- ☐ melko usein
- ☐ harvoin
- ☐ ei koskaan

**b.värillä, joka huuhtoutuu vasta usean pesun jälkeen**

- ☐ usein
- ☐ melko usein
- ☐ harvoin
- ☐ ei koskaan

**c.pysyvällä värillä, joka ei lähde pesemällä**

- ☐ usein
- ☐ melko usein
- ☐ harvoin
- ☐ ei koskaan

**d.ennen värjäystä hiuksesi valkaistiin**

- ☐ usein
- ☐ melko usein
- ☐ harvoin
- ☐ ei koskaan

**e.vain osa hiuksista värjättiin eli tehtiin raitoja**

- ☐ usein
- ☐ melko usein
- ☐ harvoin
- ☐ ei koskaan

**60. Seuraavassa luetellaan joukko tapahtumia, joita elämässä voi sattua. Jos tapahtuma on sattunut sinulle, vastaa, milloin se on sattunut. Vastaa myös, kuinka raskas koettelemus tapahtuma on ollut merkitsemällä varattuun tilaan numero ohjeen mukaan.**

| | Tapahtuma sattui: ei ollenkaan | viimeisen kahden vuoden aikana | Viimeisen viiden vuoden aikana | aikaisemmin | Tapahtuman raskaus: 1 = ei kovin raskas | 2 = raskas | 3 = äärimmäisen raskas |
|---|---|---|---|---|---|---|---|
| Puolison kuolema | ☐ | ☐ | ☐ | ☐ | ☐ | ☐ | ☐ |
| Oman lapsen kuolema | ☐ | ☐ | ☐ | ☐ | ☐ | ☐ | ☐ |
| Isän kuolema | ☐ | ☐ | ☐ | ☐ | ☐ | ☐ | ☐ |
| Äidin kuolema | ☐ | ☐ | ☐ | ☐ | ☐ | ☐ | ☐ |
| Muun läheisen sukulaisen kuolema | ☐ | ☐ | ☐ | ☐ | ☐ | ☐ | ☐ |
| Hyvän ystävän kuolema | ☐ | ☐ | ☐ | ☐ | ☐ | ☐ | ☐ |
| Perheenjäsenen vakava sairastuminen | ☐ | ☐ | ☐ | ☐ | ☐ | ☐ | ☐ |
| Raskauden keskeytys (oma tai kumppanin) | ☐ | ☐ | ☐ | ☐ | ☐ | ☐ | ☐ |
| Keskenmeno (oma tai kumppanin) | ☐ | ☐ | ☐ | ☐ | ☐ | ☐ | ☐ |
| Huomattavat vaikeudet esimiehen kanssa | ☐ | ☐ | ☐ | ☐ | ☐ | ☐ | ☐ |
| Huomattavat vaikeudet työtovereiden kanssa | ☐ | ☐ | ☐ | ☐ | ☐ | ☐ | ☐ |
| Oman taloudellisen tilanteen huomattava vaikeutuminen | ☐ | ☐ | ☐ | ☐ | ☐ | ☐ | ☐ |
| Avioero, avoero tai asumusero | ☐ | ☐ | ☐ | ☐ | ☐ | ☐ | ☐ |
| Ristiriitojen huomattava lisääntyminen avo- tai aviopuolison kanssa (ei ero) | ☐ | ☐ | ☐ | ☐ | ☐ | ☐ | ☐ |
| Välien rikkoutuminen pitkäaikaisessa ystävyyssuhteessa | ☐ | ☐ | ☐ | ☐ | ☐ | ☐ | ☐ |
| Työpaikan menetys | ☐ | ☐ | ☐ | ☐ | ☐ | ☐ | ☐ |
| Eläkkeelle siirtyminen | ☐ | ☐ | ☐ | ☐ | ☐ | ☐ | ☐ |
| Puolison/kumppanin työttömyys | ☐ | ☐ | ☐ | ☐ | ☐ | ☐ | ☐ |
| Yli kolmen viikon työkyvyttömyyden aiheuttanut sairaus | ☐ | ☐ | ☐ | ☐ | ☐ | ☐ | ☐ |
| Vakava tapaturma | ☐ | ☐ | ☐ | ☐ | ☐ | ☐ | ☐ |
| Ruumiillinen tai seksuaalinen väkivalta | ☐ | ☐ | ☐ | ☐ | ☐ | ☐ | ☐ |
| Henkinen väkivalta | ☐ | ☐ | ☐ | ☐ | ☐ | ☐ | ☐ |



9424352256

**61. Onko sinun elämässäsi sattunut jokin huomattava myönteinen kokemus tai tapahtuma?**

| | ei ollen-kaan | viimeisen kahden vuoden aikana | Viimeisen viiden vuoden aikana | aikai-semmin |
|---|---|---|---|---|
| perhe-elämässä | ☐ | ☐ | ☐ | ☐ |
| työelämässä | ☐ | ☐ | ☐ | ☐ |
| ihmissuhteissa | ☐ | ☐ | ☐ | ☐ |
| hengellisessä elämässä | ☐ | ☐ | ☐ | ☐ |
| taloudellisessa tilanteessa | ☐ | ☐ | ☐ | ☐ |
| asumisessa | ☐ | ☐ | ☐ | ☐ |
| harrastuksissa | ☐ | ☐ | ☐ | ☐ |
| rakastuminen | ☐ | ☐ | ☐ | ☐ |

KIITÄMME VAIVANNÄÖSTÄSI!

Pyydämme Sinua vielä ystävällisesti tarkistamaan, että olet vastannut kaikkiin kysymyksiin annettujen ohjeiden mukaisesti.

HUOMAA! Henkilöllisyytesi ja tietosi pidetään täysin salassa. Saatuja tutkimustuloksia ei tarkastella henkilöittäin, vaan niitä käsitellään taulukoituina. Nimesi ei tule esiin missään vaiheessa.
