# Supplementary material for: Does Hair Dye Use Increase the Risk of Breast Cancer? A Population-Based Case-Control Study of Finnish Women
Source: PLoS One. 2015 Aug 11;10(8):e0135190. doi: 10.1371/journal.pone.0135190 (PMC4532449; doi:10.1371/journal.pone.0135190)
Supplement: S2 Supporting Information — (PDF) [file pone.0135190.s004.pdf]

SURVEY ”Women’s health and use of hormones”

Partial translation from the original survey (from Finnish to English). For informational use only.

Question no. 1. Have you ever been pregnant?

YES
NO

Question no. 2. How many children have you given birth to?

ONE
TWO
THREE – FOUR
FIVE – SIX
SEVEN OR MORE

Question no. 7. Have you ever used hormonal contraceptives, such as oral contraceptive pills, contraceptive patches, contraceptive injections and/or contraceptive implant for contraceptive purpose?

YES
NO. Move to question no. 10

Question no. 12. How long in total have you used the above mentioned hormonal contraceptives for contraceptive purposes or as treatment for missing, painful or irregular menstruation or acne? (Try to estimate the total duration of all using periods combined)

I HAVE NEVER USED. Move to question no. 15.
LESS THAN 1 MONTH
1 MONTH – 6 MONTHS
OVER 6 MONTHS BUT LESS THAN 2 YEARS
2 - 4 YEARS
OVER 4 YEARS – 8 YEARS
OVER 8 YEARS – 12 YEARS
OVER 12 YEARS – 16 YEARS
OVER 16 YEARS – 20 YEARS
OVER 20 YEARS – 25 YEARS
OVER 25 YEARS

Question no. 17. How old were you when you first started menstruating?

I HAVE NEVER HAD PERIODS
12 YEARS OLD OR YOUNGER
13 YEARS OLD
14 YEARS OLD
15 YEARS OLD
16 YEARS OLD

17 YEARS OLD
18 YEARS OLD
19 YEARS OLD
20 YEARS OLD
21 YEARS OLD
22 YEARS OLD OR OLDER
I DON'T KNOW

Question no. 19. Have you ever received hormonal replacement therapy for menopausal symptoms or due to oophorectomy (removal of the ovaries)?

YES
NO. Move to question no. 25.
I DON'T KNOW

Question no. 20. How old were you when the hormone replacement therapy started?

40 YEARS OR YOUNGER
41-45 YEARS OLD
46-50 YEARS OLD
51-55 YEARS OLD
56-60 YEARS OLD
61-65 YEARS OLD
66 YEARS OR OLDER
I DON'T KNOW

Question no. 22. How old were you when you stopped the hormone replacement therapy?

40 YEARS OR YOUNGER
41-45 YEARS OLD
46-50 YEARS OLD
51-55 YEARS OLD
56-60 YEARS OLD
61-65 YEARS OLD
66-70 YEARS OLD
71-75 YEARS OLD
76 YEARS OR OLDER

Question no. 23. How long in total have you used hormone replacement therapy? (Try to estimate the total duration of all using periods combined)

LESS THAN 1 MONTH
1 MONTH – 6 MONTHS
OVER 6 MONTHS BUT LESS THAN 2 YEARS
2 - 4 YEARS
OVER 4 YEARS – 8 YEARS
OVER 8 YEARS – 12 YEARS
OVER 12 YEARS – 16 YEARS

OVER 16 YEARS – 20 YEARS
OVER 20 YEARS – 25 YEARS
OVER 25 YEARS

Question no. 41. Has your mother, sister or daughter ever been diagnosed with breast cancer?

NO. Move to question no. 43.
YES

Question no. 43. What is your marital status?

SINGLE
MARRIED OR COMMON-LAW-MARRIAGE
DIVORCED OR SEPARATED
WIDOWED

Question no. 44. What is your basic education? (Please choose only one option)

NO BASIC EDUCATION
PRIMARY SCHOOL/ELEMENTARY SCHOOL
COMPREHENSIVE SCHOOL
MATRICULATION EXAMINATION (HIGH SCHOOL)
POLYTECHNIC OR SIMILAR
UNIVERSITY-LEVEL DEGREE

Question no. 47. Have you ever smoked cigarettes regularly (daily or almost daily)?

NO. Move to question no. 53.
YES

Question no. 53. How often do you consume alcoholic drinks?

NEVER
ONLY IN SPECIAL OCCASIONS
IN THREE DAYS PER WEEK OR LESS
IN FOUR DAYS PER WEEK OR MORE

Question no. 57. Have you ever or have you ever had your hair dyed?

NEVER. Move to question no. 60.
1-2 TIMES
3-9 TIMES
10-34 TIMES
35-89 TIMES
≥ 90 TIMES

Question no. 58. At what age did you dye or had your hair dyed for the first time?

AT UNDER 20 YEARS OF AGE
AT 20-29 YEARS OF AGE

AT 30-39 YEARS OF AGE
AT 40 YEARS OF AGE OF OLDER

Question no. 59. Try to remember the way you hair was dyed.

A. *Using a color that washes off at first wash*
   OFTEN
   QUITE OFTEN
   RARELY
   NEVER
B. *Using a color that washes off after several washes*
   OFTEN
   QUITE OFTEN
   RARELY
   NEVER
C. *Using a color that does not wash off*
   OFTEN
   QUITE OFTEN
   RARELY
   NEVER
D. *The hair was bleached before dyeing*
   OFTEN
   QUITE OFTEN
   RARELY
   NEVER
E. *Only a part of the hair was dyed, i.e. it was highlighted/low-lighted*
   OFTEN
   QUITE OFTEN
   RARELY
   NEVER
