# Supplementary material for: Does Hair Dye Use Increase the Risk of Breast Cancer? A Population-Based Case-Control Study of Finnish Women
Source: PLoS One. 2015 Aug 11;10(8):e0135190. doi: 10.1371/journal.pone.0135190 (PMC4532449; doi:10.1371/journal.pone.0135190)
Supplement: S1 Appendix — (DOCX) [file pone.0135190.s005.docx]

**S1 Appendix.** Results from a deterministic sensitivity analysis

| **HAIR DYE USE,** Observed OR: 1.15 (1.06- 1.26) | | | |
| --- | --- | --- | --- |
| **Differential misclassification** of hair dye use as an exposure | | | |
| **Priors:** Sensitivity, cases: **90%** Specificity, cases: **80%** | **Priors:** Sensitivity, controls: **90%** Specificity, controls: **90%** | | BIAS-ADJUSTED ODDS RATIO (PERCENT BIAS): **2.50 (-54%)** |
| **Non-response bias,** with respect to hair dye use | | | |
| **Priors**: Exposed, cases: **0.80** Unexposed, cases: **0.60** | **Priors:** Exposed, controls: **0.60** Unexposed, controls: **0.50** | | BIAS-ADJUSTED ODDS RATIO (PERCENT BIAS): **1.04 (11%)** |
| **Uncontrolled confounding** (socio-economic status) | | | |
| Exposed (high education): **12%** Unexposed (lower education): **88%** Relative risk between high education and breast cancer: **1.36** | | BIAS-ADJUSTED ODDS RATIO (PERCENT BIAS): **1.46 (-21%)** | |
